# Oral manifestations serve as potential signs of ulcerative colitis: A review

**DOI:** 10.3389/fimmu.2022.1013900

**Published:** 2022-09-29

**Authors:** Chunyu Li, Yuqi Wu, Yulang Xie, You Zhang, Sixin Jiang, Jiongke Wang, Xiaobo Luo, Qianming Chen

**Affiliations:** State Key Laboratory of Oral Diseases, National Clinical Research Center for Oral Diseases, Chinese Academy of Medical Sciences Research Unit of Oral Carcinogenesis and Management, West China Hospital of Stomatology, Sichuan University, Chengdu, China

**Keywords:** ulcerative colitis, inflammatory bowel disease, oral manifestation, immune dysfunction, pyostomatitis vegetans

## Abstract

As an immune dysregulation-related disease, although ulcerative colitis (UC) primarily affects the intestinal tract, extraintestinal manifestations of the disease are evident, particularly in the oral cavity. Herein, we have reviewed the various oral presentations, potential pathogenesis, and treatment of oral lesions related to UC. The oral manifestations of UC include specific and nonspecific manifestations, with the former including pyostomatitis vegetans and the latter encompassing recurrent aphthous ulcers, atrophic glossitis, burning mouth syndrome, angular cheilitis, dry mouth, taste change, halitosis, and periodontitis. Although the aetiology of UC has not been fully determined, the factors leading to its development include immune system dysregulation, dysbiosis, and malnutrition. The principle of treating oral lesions in UC is to relieve pain, accelerate the healing of lesions, and prevent secondary infection, and the primary procedure is to control intestinal diseases. Systemic corticosteroids are the preferred treatment options, besides, topical and systemic administration combined with dietary guidance can also be applied. Oral manifestations of UC might accompany or precede the diagnosis of UC, albeit with the absence of intestinal symptoms; therefore, oral lesions, especially pyostomatitis vegetans, recurrent aphthous ulcer and periodontitis, could be used as good mucocutaneous signs to judge the occurrence and severity of UC, thus facilitating the early diagnosis and treatment of UC and avoiding severe consequences, such as colon cancer.

## Introduction

Ulcerative colitis (UC) is a chronic inflammatory disease of the gastrointestinal tract. Along with Crohn’s disease (CD), UC is one major type of the inflammatory bowel disease (IBD) family. Although its aetiology has not been completely determined, the factors contributing to disease development include genetic predisposition, environmental factors, immune system dysregulation, dysbiosis, and malnutrition (1, 2), of which immune dysfunction ranks as one of the most dominant aetiologies. Numerous studies have suggested that ulcerative colitis is a modified T-helper (Th) 2 disease, in which the innate immune system initiates the inflammatory events, with the adaptive immune system perpetuating the inflammatory cascade (3, 4). Similarly, another study has reported the differences in mucosal and systemic immune profiles between early and late stage disease in patients with active UC (5). Specifically, a transition from a Th1- to a Th2-driven state is revealed in the intestine of patients with UC in the late stage of disease (5).

The highest reported annual incidences of UC are 24.3, 19.2 and 6.3 per 100 000 persons in Europe, North America, and Asia together with the Middle East (6), respectively. The female-to-male ratio ranges from 0.51 to 1.58 for the incidence of UC, suggesting that the diagnosis of UC is not sex-specific (6). The peak age in most UC studies was between 20 and 40 years of age, with 51.1% of studies reporting the highest incidences of UC among people aged 20–29 years (6). The current mortality rate of patients with UC is close to or slightly higher than that of the general population (7, 8), implying the potential threat of UC. Moreover, patients with long-term UC are more likely to develop colorectal cancer than healthy controls (9). The more extensive and active the disease, the poorer the prognosis in patients with UC (10).

Common clinical features of UC consist of abdominal pain, diarrhoea, rectal bleeding, and other gastrointestinal symptoms (11). Currently, the diagnosis of UC is based on typical clinical symptoms and histological and endoscopic evidences (12), among which colonoscopy combined with biopsy is regarded as the gold standard. Specifically, endoscopy might reveal ulceration, exudate, fragility, granular mucosa, and loss of a typical vascular pattern (13, 14). However, patients with early intestinal discomfort might not be willing to undergo endoscopy. Other than these frequent symptoms of the gastrointestinal tract, 6–40% of patients suspected of having IBD might also present with various extraintestinal manifestations. Notably, the occurrence of extraintestinal manifestations may precede the ultimate diagnosis of IBD (15–18).

A substantial proportion of patients with UC may exhibit lesions within the oral cavity and perioral skin, which can occur prior to, or parallel with UC activity (18). Lesions in the oral cavity harbour innate superiority over other sites for the early detection of UC. First, oral lesions are easy to observe and examine, thus facilitating the early diagnosis of the intestinal disease; second, some oral lesions related to UC that require pathological diagnosis, such as pyostomatitis vegetans (PSV), exhibit the advantage of being observation-intuitive and biopsy-convenient compared with other sites (19). Thus, oral manifestations could serve as good cutaneous signs of the disease severity of UC, and recognition of these signs could contribute to the early detection of IBD (20). This article reviews the various oral presentations, potential pathogenesis, and treatment of oral lesions related to UC, providing guidance for clinicians in managing, diagnosing, and treating these challenging oral manifestations.

### Oral manifestations of UC

UC may be characterised by a series of specific and nonspecific oral lesions, as described in detail below (**Table 1**).

**Table 1:** Specific and nonspecific oral manifestations in patients with UC.

Oral diseases	Manifestations	Disease specificity	Treatment
Pyostomatitis vegetans	Miliary abscess and pustular lesions with white or yellow contents Erythematous and edematous mucosal base “Snail track” ulcers	Specific	Drug therapy: Topical antiseptic mouthwashes (chlorhexidine), corticosteroids (triamcinolone acetonide paste or betamethasone mouthwash), systemic steroid therapy (21, 22)
Recurrent aphthous ulcer	Recurrent bouts of one or more shallow, rounded, or ovoid painful ulcers Clear boundaries, red and slightly raised margin, and covered by yellow or white pseudomembrane Minor RAU are the most common type in UC	Not specific	Drug therapy: Topical steroids, antiseptic mouthwash, nonsteroidal anti-inflammatory pastes (23) Non-drug therapy: Laser therapy (24)
Atrophic glossitis	Glossy tongue appearance with red background Painful, burning sensation of oral mucosa and dry mouth	Not specific	Drug therapy: Vitamin and iron supplements (25)
Burning mouth syndrome	Burning sensation of the oral mucosa Dry mouth and taste disturbances	Not specific	Drug therapy: Anticonvulsants, antidepressants, phytomedicines, saliva substitute (26) Non-drug therapy: Food supplements, lower-level laser therapy, transcranial magnetic stimulation, oral appliances, cognitive behavioural therapy (26)
Angular cheilitis	Erythema, scaling, rhagades, ulcerations, and crusting of the lip corners along with the adjacent skin	Not specific	Drug therapy: Vitamin supplements, 5-ASA mouthwashes, topical steroids (1% hydrocortisone), intra-lesional steroids (27)
Taste change	Taste change	Not specific	Drug therapy: Iron, zinc, or vitamin supplements (28)
Halitosis	Halitosis	Not specific	Drug therapy: Topical mouthwash (29) Non-drug therapy: Tongue brushing (29)
Periodontitis	More severe gingival bleeding, fewer teeth, greater pocket probing depth, and higher frequency of sites with clinical attachment loss	Not specific	Drug therapy: Antibiotics (30) Non-drug therapy: Supragingival scaling and root planning, periodontal surgery (30)

UC, Ulcerative colitis; RAU, Recurrent aphthous ulcer; ASA, aminosalicylic acid.

#### Specific oral lesions

Pyostomatitis vegetans is a rare chronic mucocutaneous inflammatory disease associated with IBD and remarkably associated with UC. Thus, PSV is regarded as a highly specific marker of UC (27, 31). Lourenço et al. reported that one patient who was initially diagnosed with mild colitis accompanied by PSV, had typical features of UC on colonoscopy 2 years later (20). A recent study indicated that the subsequent onset of PSV was observed in a patient with UC upon aggravation of gastrointestinal symptoms, suggesting that PSV may indicate active or aggravated UC (32). Moreover, a patient with active UC presented with PSV after receiving an initial coronavirus disease 2019 (COVID-19) vaccination, implying the potential immunological link between the onset of PSV and UC, which might have been triggered by the vaccination (33).

**Figure 1:**
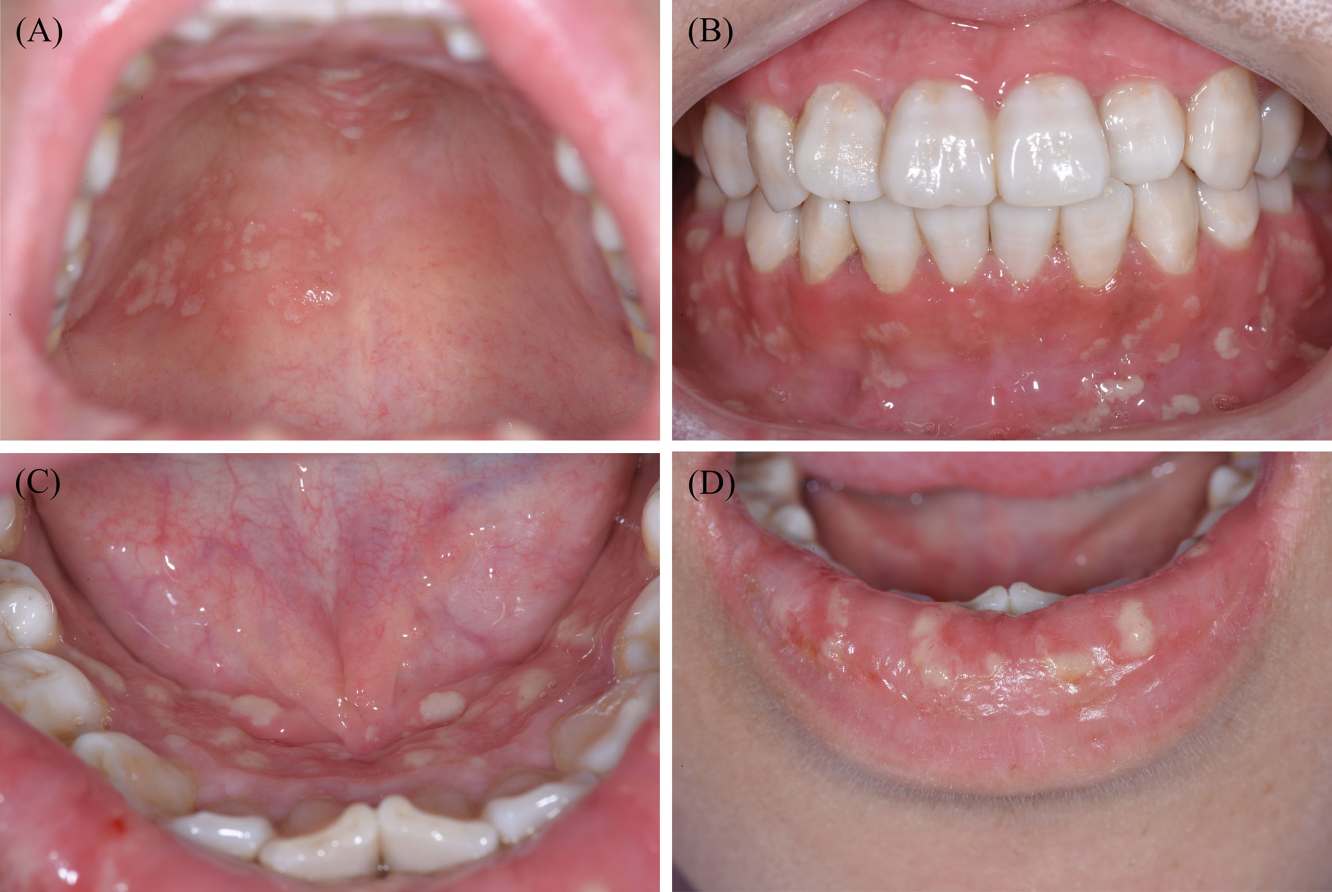
Typical clinical features of one patient with PSV in our clinic. Widespread yellow or white pustular lesions as well as its secondary ulcers were observed on the palate **(A)**, the labial gingivae **(B)**, the anterior floor of mouth **(C)**, and the lower lip **(D)** of the patient. PSV, Pyostomatitis vegetans.

As for the clinical characteristics, PSV tends to be more prevalent in patients aged between 20 and 59 years, among which men have a higher incidence than women (34, 35). Distinct clinical manifestations of PSV include miliary abscesses and pustular lesions, with white or yellow contents accompanied by erythematous and oedematous mucosal bases. Pustular lesions are prone to rupture, resulting in erosions and a characteristic ulceration resembling the morphology of the ‘snail track’ (31, 36, 37). Oral lesions predominantly involve the lips, gingiva, and buccal mucosa, although pustules may occur in the entire oral cavity (**Figure 1**). Patients may experience fever, localised pain, or enlarged and tender submandibular lymph nodes (38). In addition, the principal histological features of PSV are intraepithelial and subepithelial microabscesses, accompanied by infiltration of neutrophils and eosinophils. Furthermore, hyperkeratosis, acanthosis, and focal acantholysis may be present (20, 35).

The diagnosis of PSV is based on clinical manifestations, including concurrent IBD, peripheral eosinophilia, and histological characteristics (20, 38). Although the pathogenesis of PSV is undetermined, it is hypothesised that the abnormal immune response to unidentified factors or the presence of cross-reactive antigens in the bowel and skin leads to these secondary mucocutaneous manifestations (38). In general, PSV can resolve if the underlying UC is sufficiently controlled; however, topical corticosteroids or tacrolimus ointment can be applied to oral lesions (19, 39, 40).

#### Nonspecific oral lesions

Patients with active UC may be at a higher risk of experiencing more oral diseases that negatively affect their quality of life than those without active UC (41). Nonspecific oral lesions in UC are observed to be more prevalent than specific lesions (37), including recurrent aphthous ulcer (RAU), atrophic glossitis (AG), burning mouth syndrome (BMS), angular cheilitis (AC), dry mouth, taste change, halitosis, and periodontitis.

##### Recurrent aphthous ulcer

The most common oral mucosal lesions which occur in patients with UC are RAU (42, 43). The clinical manifestations of RAU associated with UC appear to be similar to those of RAU in the general population. It is characterised by the recurrence of one or more shallow round or ovoid ulcers with clear boundaries, surrounded by red and slightly raised margins, and covered by yellow or white pseudomembranes. Ulcers are usually painful and affect mastication as well as speech (31). Recurrent aphthous ulcers tends to involve the buccal mucosa, labial mucosa, tongue, soft palate, and other non-masticatory mucosa (44).

Currently, three clinical presentations of RAU are recognised: minor, major, and herpetiform, of which the minor type is the most frequent type found in patients with UC, presenting as small and superficial round or oval ulcers that are similar to the ulcers in the colon (45–47).

Scholars have reached controversial conclusions regarding the association between RAU and UC. One study reported that the frequency of RAU in 105 patients with UC, aged from 17 to 82 years, was 18.1% among those with extraintestinal manifestations, and 27.8% of patients were diagnosed with RAU before the diagnosis of IBD among those presenting with oral manifestations (18). Similarly, another team have showed that the frequency of aphthous ulcers in 50 patients with UC was 20% (42). Another study reported that the frequency of oral manifestations among 119 patients with UC, aged from 19 to 77 years, was 29.4%, and 15.1% of patients were diagnosed as RAU, accounting for more than half of the oral manifestations (48). Besides, Khozeimeh and colleagues showed that RAU could appear 1–3 years before the diagnosis of UC (48). Furthermore, Habashneh et al. pointed out that the incidence of deep oral ulceration in patients with UC was markedly higher than in participants without UC (*p*=0.004) (49). Moreover, Laranjeira et al. found that 35.3% of patients complained about the occurrence of RAU within the active stage of IBD, compared with 4.2% in the remissive phase of IBD (50). Similarly, the exacerbation of RAU was reported during UC recurrence (36); Elahi et al. found the frequency of RAU in patients with severe UC was 46%, 18% in patients with moderate UC, and 5% in patients with mild UC (42). All studies suggested that the assessment of RAU severity might be employed as an indicator of UC relapse and recurrence. However, a few studies have not shown a significant relationship between UC disease activity and RAU frequency (16, 41); for instance, one study found that the frequency of RAU in patients with active UC was 4.1%, while that in patients with inactive UC was 3%,with no statistical difference indicated (16). Another research implied that the frequency of RAU in patients with active UC was 21.4%, while that in patients with inactive UC was 29.7%, with no statistical difference suggested (41). More well-designed multi-centre studies are warranted to validate the association between RAU and UC.

##### Other oral mucosal lesions associated with ulcerative colitis

Atrophic glossitis or smooth tongue is an atrophic change of the tongue mucosa which might exhibit a glossy appearance with a red background, caused by atrophy of the filiform papillae (51). Histologically, epithelial atrophy and chronic inflammation of the subepithelial connective tissue are key features of AG (52). The main symptoms reported in previous research were pain, burning sensation in the oral mucosa, and dry mouth. Atrophic glossitis is more common in CD than in UC; and the incidence of AG in patients with UC along with its association with disease activity remains unclear (25). Further studies are required to fill this gap in the field.

Patients with BMS mainly present with a burning sensation of the oral mucosa, sometimes accompanied by dry mouth, taste disturbances, and other discomfort. Usually, the burning sensation is bilateral, and most commonly involves the tongue, followed by the labial mucosa and anterior hard palate (53, 54). Goldinova et al. found that 9.8% of patients with UC suffered from BMS, compared with an absence of BMS in the control group without UC. Moreover, BMS was found in 14.3% and 8.1% of patients with UC in active or inactive phase of the intestinal disease, respectively (41). The authors also demonstrated that subjective perception of dry mouth is related to disease activity of UC, while no correlation was found with the objective salivary secretion rate (41).

Angular cheilitis is characterised by erythema, scaling, rhagades, ulcerations, and crusting of the lip corners and adjacent skin (55). Klichowska-Palonka et al. reported that the incidence of AC in paediatric patients with UC is approximately 12.5% (56). Furthermore, Goldinova and colleagues found that 21.6% of patients with UC had AC, compared with none in the control group without UC (41). Thus, AC may serve as another associated condition of UC.

Additionally, Melis et al. reported that patients with IBD had impaired salty, sweet, bitter, umami, and fat tastes but an increased sour taste (28). Elahi et al. found that 40% of patients with UC presented with taste change, while 68% of patients with UC had halitosis, which demonstrated a statistically significantly difference from the control set (42). Katz et al. included 20 patients with UC, 10 of whom were in the active disease stage. The incidence of halitosis was notably higher in the active UC group than in the control group (50% versus 10%) (57). Additionally, another study indicated that in the presence of the perinuclear antineutrophil cytoplasmic antibody, immunoglobulins A and G, anti-*Saccharomyces cerevisiae* antibodies, antibodies to *Escherichia coli* outer membrane porin C, anti-flagellin antibody, fragments of *Pseudomonas fluorescens* bacterial DNA, and other serological immune markers (58), further investigation with colonoscopy might be prompted to exclude the diagnosis of IBD.

##### Periodontitis

Periodontitis is one of the most common oral diseases that is closely related to systemic diseases; which is inseparable from systemic and local immune dysregulation (59). To date, various studies have demonstrated inconsistent results on the relationship between periodontitis and the occurrence of UC.

Four meta-analyses revealed that, compared to healthy controls, patients with UC were significantly more likely to experience periodontitis, have fewer teeth, greater pocket probing depth, and higher frequency of sites with clinical attachment loss (CAL) >3mm (60–63). Habashneh et al. highlighted that patients with UC had more severe and significantly higher risk of developing periodontitis than patients without IBD (49). Another study found that, compared with healthy control subjects, IBD patients exhibited more severe gingival bleeding (*p*<0.01), periodontitis (*p*=0.04) and higher CAL (*p*<0.01) (64). Vavricka et al. had reached similar conclusions. Moreover, IBD, as a spectrum of diseases, is also regarded as a significant risk factor for periodontitis (OR=3.35) (65). Koutsochristou et al. reported that there was an increased incidence of periodontal disease in children and adolescents with IBD, although the oral hygiene indicators were comparable to controls (66).

Contrastingly, Grössner et al. found no significant differences regarding the periodontal findings for patients with or without IBD (67). Tan et al. reported that, compared with non-IBD patients, the DPSI (Dutch Periodontal Screening Index) scores were not significantly increased in UC patients (68).

Taken together, the manifestations of PSV, RAU, AG, BMS, AC, dry mouth, taste change, halitosis and periodontitis may serve as a clue to the diagnosis of UC (18, 20, 25, 41, 42, 56, 61); Among these, RAU and periodontitis might serve as a strong sign of latent UC through assessing these relevant studies using Grading of Recommendations Assessment, Development and Evaluation (GRADE) standard (69), and PSV, as a rare disease, may represent the relatively specific sign of UC to some extent (**Table 2**). Therefore, when patients present with these oral manifestations, they should be screened for UC by enquiring specifically about potential UC-related gastrointestinal symptoms upon history-taking. If necessary, relevant antibodies tests, and endoscopy as well as biopsy should be considered by gastroenterologists to determine the presence of UC and potential intestinal cancer.

**Table 2:** Evaluation of the level of evidence-based medicine of these research findings.

References	Oral manifestations	Research type	GRADE classification
Vavricka 2011 (16)	RAU	Cohort study	Moderate
Vavricka 2015 (18)	RAU	Cohort study	Moderate
Kamal 2020 (19)	PSV	Case report	Very low
Lourenço 2010 (20)	PSV	Case reports	Very low
Zeng 2022 (32)	PSV	Case report	Very low
Hou 2022 (33)	PSV	Case report	Very low
Ruiz-Roca 2005 (35)	PSV	Case report	Very low
Kumar2018 (36)	RAU	Case-control study	Low
Yasuda 2008 (39)	PSV	Case report	Very low
Werchniak 2005 (40)	PSV	Case report	Very low
Goldinova 2020 (41)	RAU, BMS, AC, dry mouth	Cohort study	Low
Elahi 2012 (42)	RAU, taste change, halitosis	Case-control study	Low
Khozeimeh 2021 (48)	RAU	Cross-sectional study	Low
Habashneh 2012 (49)	RAU, periodontitis	Case-control study	Low
Laranjeira 2015 (50)	RAU	Case-control study	Low
Klichowska-Palonka 2021 (56)	AC	Case-control study	Low
Melis 2020 (28)	Taste change	Case-control study	Low
Katz 2003 (57)	Halitosis	Case-control study	Low
She 2020 (60)	Periodontitis	Meta analysis	Moderate
Zhang 2021 (61)	Periodontitis	Meta analysis	Moderate
Lorenzo-Pouso 2021 (62)	Periodontitis	Meta analysis	Moderate
Papageorgiou 2017 (63)	Periodontitis	Meta analysis	Moderate
Schmidt 2018 (64)	Periodontitis	Case-control study	Low
Vavricka 2013 (65)	Periodontitis	Cohort study	Moderate
Koutsochristou 2015 (66)	Periodontitis	Case-control study	Low
Grössner-Schreiber 2006 (67)	Periodontitis	Case-control study	Low
Tan 2021 (68)	Periodontitis	Case-control study	Low

GRADE, Grading of Recommendations Assessment, Development and Evaluation; PSV, pyostomatitis vegetans; RAU, Recurrent aphthous ulcer; BMS, burning mouth syndrome; AC, angular cheilitis.

### Pathogenesis

**Figure 2:**
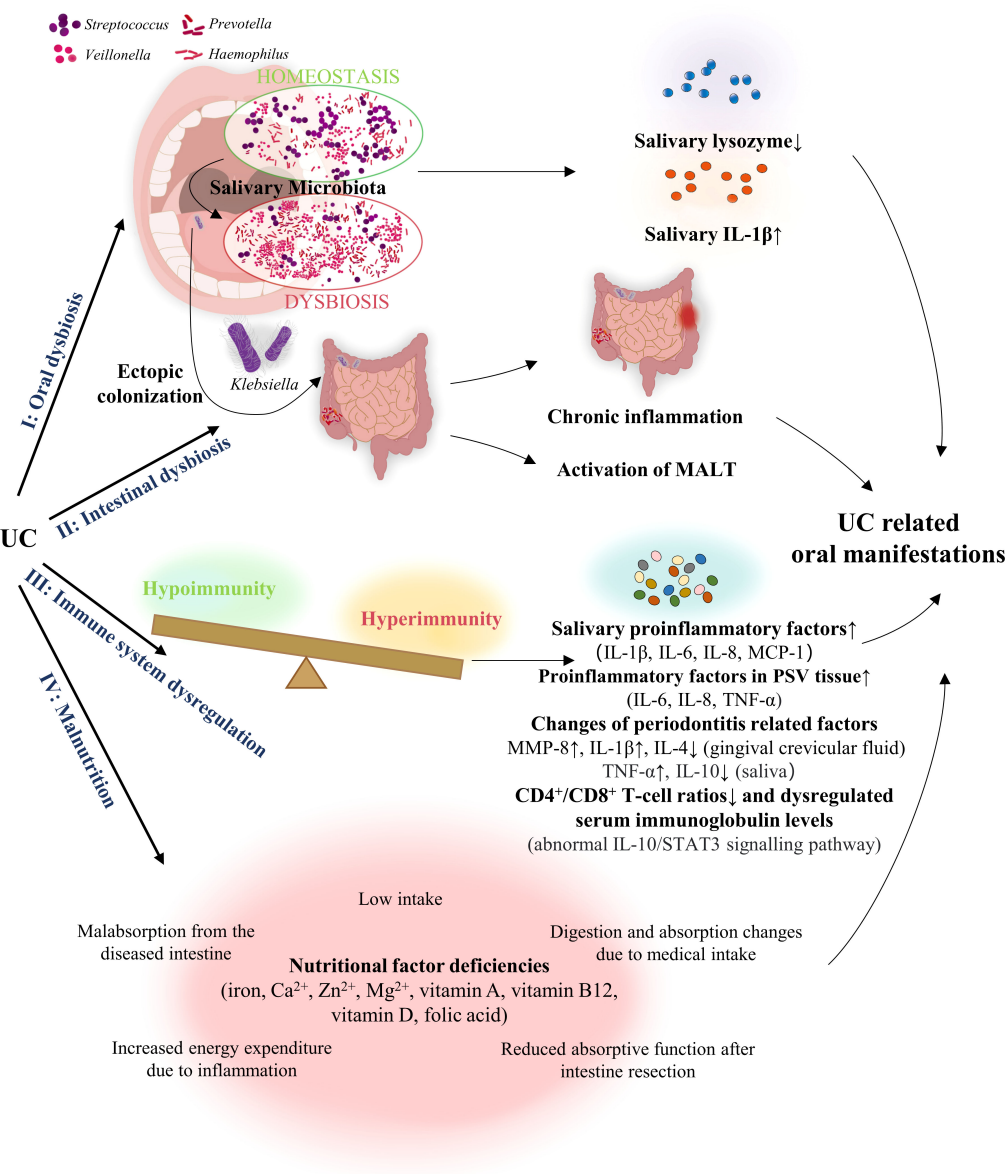
Pathogenesis of the oral manifestations associated with UC. UC, Ulcerative colitis; MALT, mucosa-associated lymphatic tissue; MCP, monocyte chemoattractant protein; IL, interleukin; MMP-8, matrix-metalloproteinase 8; TNF, tumour necrosis factor; PSV, Pyostomatitis vegetans.

The exact pathologic mechanism of the oral manifestations associated with UC is still uncharacterised but is potentially related to dysbiosis, immune system dysregulation, and malnutrition (**Figure 2**).

#### Dysbiosis

Several studies have indicated a possible correlation between dysbiosis of the gut, and oral microbiota, with the latter causing UC-related oral manifestations. Said et al. have demonstrated that oral dysbiosis, manifesting as a relative abundance of *Streptococcus*, *Haemophilus*, *Prevotella*, and *Veillonella* in the oral cavity, was closely related to the inflammatory response triggered by lower saliva lysozyme and increased interleukin (IL)-1β levels which might be related to dysbiosis of the gut microbiota (70). In addition, Xun et al. found that *Streptococcus*, *Corynebacterium*, *Lautropia*, *Acinetobacter*, and *Cardiobacterium* were observably enriched in the oral cavity of UC patients, while *Anaerovorax*, *Porphyromonas*, *Prevotella*, *Catonella*, *Oribacterium*, and *Peptostreptococcus* were significantly eliminated in the oral cavity of these patients. Simultaneously, their study also showed that the biosynthesis and transport of substances that enhance oxidative stress and virulence, bacterial violence, enzyme families activity, and the frequency of apoptosis in the oral region were increased in patients with UC, thus suggesting that the presence of both oral dysbiosis and functional disorders might be associated with UC (71).

With regards to oral dysbiosis, Molinero et al. found that *Proteobacteria* and *Neisseriaceae* in the saliva were higher in the UC group, while *Peptostreptococcaceae*, *Atopobiaceae*, *Lachnospiraceae*, and *Ruminococcaceae* were significantly reduced. They also reported that the *Staphylococcus* species and its four differential species or phylotypes were only present in UC patients, and not in the control group (72). Further, changes in salivary lysozyme levels may be associated with gut dysbiosis and is responsible for the resulting periodontitis (73). Therefore, the secondary inflammatory responses induced by UC play a critical role in the development of oral manifestations such as periodontitis (74).

Additionally, the resemblance between oral and gut microbiota might be another factor bridging UC and oral lesions. One study demonstrated that *Enterobacteriaceae* residing in saliva, particularly *Klebsiella*, were considered as potent Th1 inducers in the gut that might trigger pathogenic immune responses during ectopic intestinal colonisation (75). The inflammatory state of IBD may render the intestine more permissive to aerotolerant oral-derived bacteria than a steady-state intestine, and the continuous colonisation of oral bacteria may contribute to intestinal microbiota dysbiosis and chronic inflammation, thus intensifying the UC. Moreover, several studies have reported that *Enterobacteriaceae*, including *E. coli* and *Klebsiella*, which mainly appear in the gut, also reside in the oral cavity of humans (76, 77). Rautava et al. found that dysbiosis in murine models of colitis was related to changes in the composition of the bacteria present in saliva and in the oral cavity. Such changes in the oral microbiota may be related to the aetiology of the oral mucosal pathologies in patients with IBD (78). Thus, the oral microbiota may serve as a link between IBD types such as UC, and the related oral manifestations.

Moreover, intestinal dysbiosis has been reported to cause a chronic inflammatory state and mucosa-associated lymphatic tissue (MALT) activation in the intestine, which leads to extraintestinal pathologies (79–81). On this basis, Cappello et al. hypothesised that oral aphthous-like ulcers in patients with IBD could be the result of concomitant intestinal dysbiosis and other events, e.g., microtraumas of the oral mucosa (82). Adjuvant therapy with probiotics has been shown to be effective in treating aphthous-like ulcers, serving as proof for this hypothesis (82).

In summary, dysbiosis of microbiota in UC may be correlated with that in the oral cavity, and secondary inflammatory responses within the gut might be responsible for these oral manifestations.

#### Immune system dysregulation

Several studies have revealed the potential relationship between UC and the oral presentation. As UC is a chronic and severe disease, low levels of IL-10 within the body are insufficient to inhibit the production of pro-inflammatory cytokines, such as IL-6, tumour necrosis factor (TNF)-α, interferon-γ, and IL-17. Thus, the resultant higher IL-17/IL-10 ratio may be a pivotal marker of disease severity and progression based on its association with intestinal and extra-intestinal features (83). Oral presentations in patients with active UC may be associated with changes in cytokine activity within the gastrointestinal tract and the oral cavity (84). For instance, Said et al. reported elevated salivary levels of IL-6, IL-8, IL-1β, and monocyte chemoattractant protein (MCP)-1 in patients with UC (70). Similarly, Aleksandra et al. demonstrated that patients with UC exhibited higher salivary IL-6 levels (85). Two studies have shown that the IL-10/STAT3 signalling pathway is associated with pediatric IBD, and IL-10 receptor (IL-10R) A together with IL-10RB mutations, responsible for the abnormal IL-10/STAT3 cascade, are common in Chinese children with IBD (86, 87). Engelhardt et al. indicated that IL-10 and IL-10R deficient patients may display RAU due to mild immunological abnormalities, including reduced CD4^+^/CD8^+^ T-cell ratios and dysregulated serum immunoglobulin levels (88). Thus, these studies suggest that IL-10 related immune abnormalities may explain the initiation of RAU subsequent to UC.

Of note, several dysregulated immune-related cytokines in saliva or oral cavity, which account for periodontitis, might be related to UC. Schmidt et al. found that, compared with healthy control subjects, patients with IBD showed higher concentration of activated matrix-metalloproteinase 8 (MMP-8) in the gingival crevicular fluid, which has been confirmed as one of the key proteases released by inflammatory cells involved in the progression of periodontitis (64). Figueredo et al. observed that the total level of IL-4, a cytokine related to generalised aggressive periodontitis (89), was significantly reduced in the gingival crevicular fluid obtained from superficial sites of patients with UC (90). Meanwhile, the study also showed markedly enhanced serum IL-18 levels in the UC group, which was positively correlated with increased IL-1β in the gingival crevicular fluid of these patients. The latter served as a contributor in the pathogenesis of periodontal disease, thus accounting for the occurrence of periodontitis in patients with UC (90). Another study similarly demonstrated an increase of IL-1β in the gingival crevicular fluid of patients with UC (91). In addition, Enver et al. found that TNF-α levels were significantly higher in the saliva of patients with UC diagnosed with periodontitis, along with a reduction of IL-10 levels (91).

Meanwhile, oral diseases might also promote UC development in an immune-dependent manner. One study indicated that bacteria contributing to periodontitis might stimulate the production of pro-inflammatory cytokines by oral epithelial cells, namely IL-6, IL-8, and TNF-α, thus resulting in the progression of UC (92).

Conversely, UC is a possible immune trigger of oral manifestations. As speculated by Brakenhoff et al., the ability of IBD to induce extra-intestinal inflammatory response is partially due to the recognition of common epitopes throughout the body. Specifically, the extraintestinal manifestations of IBD may be a broad adaptive immune response caused by local intestinal dysbiosis, leading to recognition of these epitopes in gingiva and other oral sites, causing periodontitis (93, 94). Additionally, studies have shown that immune responses to colonic bacteria dysregulation in patients with IBD may trigger T cell-mediated responses and cytokine production, thus inducing PSV (95). Ficarra et al. found that T cells would transfer to the oral mucosa under the influence of antigenic stimulation. Thereafter, the CD8^+^T lymphocytes, as well as the infiltrating macrophages and neutrophils, might induce the epithelial damage and ulceration which are prevalent in PSV (96). They also demonstrated the overexpression of IL-6, IL-8, and TNF-α in PSV, suggesting that these pro-inflammatory cytokines, which lead to the recruitment of inflammatory cells to UC lesions, may synergistically contribute to the proinflammatory pathogenesis of PSV (96). Therefore, these studies suggest that immune system dysregulation may be an important link bridging PSV and UC.

Therefore, these studies suggest that immune system dysregulation may be an important link bridging not only PSV, but also other oral manifestations, and UC.

#### Malnutrition

Approximately 23% of outpatients and 85% of hospitalised patients with UC develop malnutrition (97). Malnutrition with undernutrition is common in patients with UC due to insufficient intake, malabsorption in the diseased intestine, decreased absorptive function after intestinal resection, increased energy consumption caused by inflammation, and changes in digestion and absorption due to medication intake (98). Patients with UC might develop anaemia due to deficiencies of iron, folic acid, and vitamin B12 from a lack of nutrition (98–100). Sun et al. found that deficiencies in haemoglobin, iron, and vitamin B12 were significantly correlated with AG (101). Wu et al. showed that the incidence of oral features, including lingual varicosity, AG, dry mouth, and burning sensation of the oral mucosa, was considerably higher in patients with iron-deficiency anaemia than in those without (102). Lin et al. showed that patients with BMS have a significantly higher incidence of haemoglobin, iron, or vitamin B12 deficiency (103). Nutritional deficiencies, including deficiencies of iron and B vitamins, impede wound healing and account for 25% of all AC cases (104). Hence, patients with UC are at an increased risk of micronutrient deficiencies, including calcium, iron, vitamin A, vitamin B12, vitamin D, folic acid, magnesium, and zinc (1). Therefore, patients with UC may develop RAU, AG, BMS, AC, dry mouth, taste change, and halitosis due to nutritional deficiencies.

### Treatment strategies for UC-related oral discomfort

The purpose of treating oral lesions in UC is to relieve pain, expedite the concrescence of lesions, and prevent secondary infections (37). In most patients with oral lesions associated with UC, initial control of intestinal diseases is critical for the treatment of oral signs (20). Topical and systemic medications combined with dietary instructions may also be used. In brief, the preferred treatment involves the application of systemic corticosteroids, which are often helpful in relieving the oral manifestations of patients with UC. Immunosuppressive and biological agents have also been suggested (105).

Treatment for PSV includes topical use of antiseptic mouthwashes, such as chlorhexidine, and corticosteroids, such as betamethasone mouthwash or triamcinolone acetonide paste. Systemic steroid therapy may also be used to control lesions because of the limited efficacy of topical steroid therapy (21, 22). One case study demonstrated that for PSV lesions in patients with UC, despite the ineffectiveness of topical clobetasol propionic ointment and betamethasone gargle, the administration of dapsone (75 mg/day) successfully controlled PSV in 1 week, and all eruptions disappeared after 4 weeks (106). However, Bardasi et al. reported that oral beclomethasone was capable of gradually relieving PSV lesions (107). Interestingly, one case study indicated that although partial improvement of PSV lesions was achieved after total colectomy, new lesions appeared 1 month postoperatively. The patient’s lesion was topically treated with tacrolimus ointment, which resulted in significant improvement of PSV (39). In contrast, Kitayama et al. showed that after subtotal colectomy for UC, the prescribed medicine to control intestinal symptoms could alleviate the PSV lesion simultaneously. The patient was treated with oral mesalazine, with no recurrence of mucocutaneous or intestinal lesions at follow-up (108).

For UC-related RAU, intestinal symptoms should be initially controlled, and the treatment modality for RAU depends on the frequency and severity of oral ulcers. Given the painful presentation and inflammatory nature of RAU, it responds well to the application of topical or systemic anti-inflammatory agents, especially corticosteroids (23). Topical steroids are the first-line treatment for RAU, whereas topical anaesthetics, antiseptic mouthwash, or nonsteroidal anti-inflammatory pastes can also be utilised (109). Pereira et al. reported that in one case of UC with RAU, the RAU was initially treated with 0.05% dexamethasone mouthwash for 3 months, with the symptoms being completely resolved. Then, the patient was instructed to use 0.05% dexamethasone mouthwash whenever RAU recurred (110).

Laser therapy may also help improve pain control and promote the healing of recalcitrant RAU lesions (24). Aggarwal found that after application of low-level laser therapy for RAU, the patient’s pain score decreased immediately and remained stable for 3 follow-up days (*p*<0.001 for all time periods). Additionally, the size of the lesions after low-level laser therapy decreased significantly (*p*<0.05) (111).

AG, BMS, and AC are usually caused by anaemia and malnutrition, and iron, folic acid, and vitamin B12 supplements are required in patients with specific deficiencies (98–100). For the management of AG, vitamin and iron supplements may be effective (25). Vitamin supplements, topical steroids (1% hydrocortisone), 5-aminosalicylic acid (5-ASA) mouthwashes, and intralesional steroids may be used to treat AC (27). The primary goal of BMS therapy is to eliminate painful burning disorders. Current treatments for BMS include anticonvulsants, antidepressants, phytomedicines, food supplements, lower-level laser therapy, saliva substitutes, transcranial magnetic stimulation, oral appliances, and cognitive behavioural therapy (26). Taste change may be triggered by iron, zinc, or vitamin deficiency due to rectal bleeding and intestinal malabsorption associated with IBD or by drug therapy (28), which should be supplemented with deficient nutrients or adjusted IBD medication. Tongue brushing and application of topical mouthwash, such as 0.2% chlorhexidine, are effective in treating halitosis (29).

With regard to the UC patient with concurrent periodontitis, routine dental treatment including supragingival scaling and root planning combined with drug therapy such as antibiotics and periodontal surgery are suggested as first-line therapies (30). Further, although no direct evidence exists in terms of the relationship between the UC treatment and periodontal disease’s outcome, several findings might serve as auxiliary proof. One study indicated that patients with UC but undiagnosed periodontitis showed improvement in salivary immunoglobulin A and myeloperoxidase after the treatment against UC, suggesting that UC therapy may improve the oral host defense, which is crucial to controlling periodontitis due to its microbial aetiology (112). Another study demonstrated that the treatment of IBD with anti-TNF-α biologic agents increased the probability of periodontal healing (113).

Furthermore, for UC patients with highly resistant or intractable oral lesions that seriously affect oral feeding and quality of life, colectomy may be the ultimate solution (45). A recent study demonstrated that one patient with UC achieved complete remission of PSV immediately after total colectomy due to a final diagnosis of colon adenocarcinoma (114).

### Conclusions

Oral manifestations of UC include specific lesions, such as PSV, and nonspecific lesions, namely RAU, AG, BMS, AC, dry mouth, taste change, halitosis, and periodontitis. The pathogenesis of oral manifestations in UC may be related to dysbiosis, immune system dysregulation, and malnutrition. Management of intestinal diseases is the foremost step in the treatment of oral lesions in patients with UC. Topical and systemic medications, including systemic corticosteroids, immunosuppressive agents, and biological agents, combined with dietary instructions can also be used to treat oral signs.

Oral manifestations of UC may be the most intuitive evidence of an underlying systemic disease. Oral lesions may accompany or precede the diagnosis of UC. When oral manifestations are accompanied by abdominal pain, diarrhoea, rectal bleeding, and other intestinal symptoms, stomatologists should cooperate with gastroenterologists to actively investigate the possibility of UC. The presence of oral manifestations, such as PSV, might be a crucial indicator of UC, even in the absence of intestinal symptoms, thereby enabling treatment of the disease at an early stage to prevent more severe consequences, such as colon cancer. However, the incidence of nonspecific oral lesions in patients with UC along with its association with disease activity remains largely uncharacterised. Moreover, the exact pathologic mechanism of the oral manifestations associated with UC has yet to be thoroughly characterised. Further studies are required to fill these gaps.

## Author contributions

CL was mainly responsible for drafting and organization of the work, YW and YX collated the literature data and participated in the draft preparation, YZ and SJ conducted the literature search, JW assisted in revision of the work, XL made contributions to the conceptional design and substantively revised the work, QC provided suggestions to the conceptional design of the work and revised the work. All authors have approved the final manuscript.

## Funding

The work is funded by National Natural Science Foundation of China (81902782, 82002888, 81730030), Research Funding from West China School/Hospital of Stomatology Sichuan University (No.RCDWJS2022-16), the CAMS Innovation Fund for Medical Sciences (CIFMS, 2019-I2M-5-004), the 14th special grant from China Postdoctoral Science Foundation (2021T140484).

## Conflict of interest

The authors declare that the research was conducted in the absence of any commercial or financial relationships that could be construed as a potential conflict of interest.

## Publisher’s note

All claims expressed in this article are solely those of the authors and do not necessarily represent those of their affiliated organizations, or those of the publisher, the editors and the reviewers. Any product that may be evaluated in this article, or claim that may be made by its manufacturer, is not guaranteed or endorsed by the publisher.

## References

[1] Owczarek D Rodacki T Domagała-Rodacka R Cibor D Mach T. Diet and nutritional factors in inflammatory bowel diseases. World J Gastroenterol (2016) 22:895–905. doi: 10.3748/wjg.v22.i3.895 26811635 PMC4716043

[2] Ramos GP Papadakis KA. Mechanisms of disease: Inflammatory bowel diseases. Mayo Clinic Proc (2019) 94:155–65. doi: 10.1016/j.mayocp.2018.09.013 PMC6386158 30611442

[3] Xavier RJ Podolsky DK. Unravelling the pathogenesis of inflammatory bowel disease. Nature (2007) 448:427–34. doi: 10.1038/nature06005 17653185

[4] Ungaro R Mehandru S Allen PB Peyrin-Biroulet L Colombel J-F. Ulcerative colitis. Lancet (London England) (2017) 389:1756–70. doi: 10.1016/S0140-6736(16)32126-2 PMC6487890 27914657

[5] Mavroudis G Magnusson MK Isaksson S Sundin J Simrén M Öhman L. Mucosal and systemic immune profiles differ during early and late phases of the disease in patients with active ulcerative colitis. J Crohn's Colitis (2019) 13:1450–8. doi: 10.1093/ecco-jcc/jjz072 30946450

[6] Molodecky NA Soon IS Rabi DM Ghali WA Ferris M Chernoff G. Increasing incidence and prevalence of the inflammatory bowel diseases with time, based on systematic review. Gastroenterology (2012) 142. doi: 10.1053/j.gastro.2011.10.001 22001864

[7] Jess T Gamborg M Munkholm P Sørensen TIA. Overall and cause-specific mortality in ulcerative colitis: Meta-analysis of population-based inception cohort studies. Am J Gastroenterol (2007) 102:609–17. doi: 10.1111/j.1572-0241.2006.01000.x 17156150

[8] Manninen P Karvonen AL Huhtala H Rasmussen M Salo M Mustaniemi L. Mortality in ulcerative colitis and crohn's disease. A population-based study in Finland. J Crohn's colitis (2012) 6:524–8. doi: 10.1016/j.crohns.2011.10.009 22398058

[9] Yashiro M. Ulcerative colitis-associated colorectal cancer. World J Gastroenterol (2014) 20:16389–97. doi: 10.3748/wjg.v20.i44.16389 PMC4248182 25469007

[10] da Silva BC Lyra AC Rocha R Santana GO. Epidemiology, demographic characteristics and prognostic predictors of ulcerative colitis. World J Gastroenterol (2014) 20:9458–67. doi: 10.3748/wjg.v20.i28.9458 PMC4110577 25071340

[11] Kucharzik T Koletzko S Kannengiesser K Dignass A. Ulcerative colitis-diagnostic and therapeutic algorithms. Deutsches Arzteblatt Int (2020) 117:564–74. doi: 10.3238/arztebl.2020.0564 PMC8171548 33148393

[12] Feuerstein JD Cheifetz AS. Ulcerative colitis: Epidemiology, diagnosis, and management. Mayo Clinic Proc (2014) 89:1553–63. doi: 10.1016/j.mayocp.2014.07.002 25199861

[13] Langan RC Gotsch PB Krafczyk MA Skillinge DD. Ulcerative colitis: diagnosis and treatment. Am Family physician (2007) 76:1323–30. 18019875

[14] Fine KD Seidel RH Do K. The prevalence, anatomic distribution, and diagnosis of colonic causes of chronic diarrhea. Gastrointestinal endoscopy (2000) 51:318–26. doi: 10.1016/s0016-5107(00)70362-2 10699778

[15] Bernstein CN Blanchard JF Rawsthorne P Yu N. The prevalence of extraintestinal diseases in inflammatory bowel disease: A population-based study. Am J Gastroenterol (2001) 96:1116–22. doi: 10.1111/j.1572-0241.2001.03756.x 11316157

[16] Vavricka SR Brun L Ballabeni P Pittet V Prinz Vavricka BM Zeitz J. Frequency and risk factors for extraintestinal manifestations in the Swiss inflammatory bowel disease cohort. Am J Gastroenterol (2011) 106:110–9. doi: 10.1038/ajg.2010.343 20808297

[17] Ricart E Panaccione R Loftus EV Jr Tremaine WJ Harmsen WS Zinsmeister AR. Autoimmune disorders and extraintestinal manifestations in first-degree familial and sporadic inflammatory bowel disease: A case-control study. Inflammation Bowel Dis (2004) 10:207–14. doi: 10.1097/00054725-200405000-00005 15290913

[18] Vavricka SR Rogler G Gantenbein C Spoerri M Prinz Vavricka M Navarini AA. Chronological order of appearance of extraintestinal manifestations relative to the time of IBD diagnosis in the Swiss inflammatory bowel disease cohort. Inflammation Bowel Dis (2015) 21:1794–800. doi: 10.1097/MIB.0000000000000429 26020601

[19] Kamal N Bookwalter A Foss R Cross RK. Pyostomatitis vegetans: An unusual oral manifestation of inflammatory bowel disease. Am J Gastroenterol (2020) 115:1385. doi: 10.14309/ajg.0000000000000532 32886871

[20] Lourenço SV Hussein TP Bologna SB Sipahi AM Nico MMS. Oral manifestations of inflammatory bowel disease: A review based on the observation of six cases. J Eur Acad Dermatol Venereology JEADV (2010) 24:204–7. doi: 10.1111/j.1468-3083.2009.03304.x 19552719

[21] Trost LB McDonnell JK. Important cutaneous manifestations of inflammatory bowel disease. Postgraduate Med J (2005) 81:580–5. doi: 10.1136/pgmj.2004.031633 PMC1743347 16143688

[22] Brinkmeier T Frosch PJ. Pyodermatitis-pyostomatitis vegetans: a clinical course of two decades with response to cyclosporine and low-dose prednisolone. Acta dermato-venereologica (2001) 81:134–6. doi: 10.1080/00015550152384290 11501652

[23] Scully C Gorsky M Lozada-Nur F. The diagnosis and management of recurrent aphthous stomatitis: A consensus approach. J Am Dental Assoc (1939) (2003) 134:200–7. doi: 10.14219/jada.archive.2003.0134 12636124

[24] Saikaly SK Saikaly TS Saikaly LE. Recurrent aphthous ulceration: A review of potential causes and novel treatments. J Dermatol Treat (2018) 29:542–52. doi: 10.1080/09546634.2017.1422079 29278022

[25] Sbeit W Kadah A Mahamid M Karayanni H Mari A Tali S. Oral manifestations of inflammatory bowel disease: The neglected piece of the puzzle. Eur J Gastroenterol Hepatol (2020) 32:1422–31. doi: 10.1097/MEG.0000000000001918 32925508

[26] Tan HL Smith JG Hoffmann J Renton T. A systematic review of treatment for patients with burning mouth syndrome. Cephalalgia an Int J Headache (2022) 42:128–61. doi: 10.1177/03331024211036152 PMC8793318 34404247

[27] Lankarani KB Sivandzadeh GR Hassanpour S. Oral manifestation in inflammatory bowel disease: A review. World J Gastroenterol (2013) 19:8571–9. doi: 10.3748/wjg.v19.i46.8571 PMC3870502 24379574

[28] Melis M Mastinu M Sollai G Paduano D Chicco F Magrì S. Taste changes in patients with inflammatory bowel disease: Associations with PROP phenotypes and polymorphisms in the salivary protein, gustin and CD36 receptor genes. Nutrients (2020) 12(2):409. doi: 10.3390/nu12020409 PMC7071215 32033224

[29] Bollen CML Beikler T. Halitosis: The multidisciplinary approach. Int J Oral Sci (2012) 4:55–63. doi: 10.1038/ijos.2012.39 22722640 PMC3412664

[30] Graziani F Karapetsa D Alonso B Herrera D. Nonsurgical and surgical treatment of periodontitis: How many options for one disease? Periodontol 2000 (2017) 75:152–88. doi: 10.1111/prd.12201 28758300

[31] Field EA Allan RB. Review article: oral ulceration–aetiopathogenesis, clinical diagnosis and management in the gastrointestinal clinic. Alimentary Pharmacol Ther (2003) 18:949–62. doi: 10.1046/j.1365-2036.2003.01782.x 14616160

[32] Zeng X Hua H Hu X. Rare mucocutaneous manifestations of ulcerative colitis: A case report of pyostomatitis vegetans and sweet syndrome. Oral Surg Oral Med Oral Pathol Oral Radiol (2022) S2212-4403:01016-1. doi: 10.1016/j.oooo.2022.06.004 35987735

[33] Hou P-C Huang H-Y Lee JY-Y Hsu C-K. Pyostomatitis vegetans following coronavirus disease 2019 vaccination in a patient with ulcerative colitis. J Dermatol (2022) 49:e285–6. doi: 10.1111/1346-8138.16409 PMC9347529 35491619

[34] Hegarty AM Barrett AW Scully C. Pyostomatitis vegetans. Clin Exp Dermatol (2004) 29:1–7. doi: 10.1111/j.1365-2230.2004.01438.x 14723710

[35] Ruiz-Roca JA Berini-Aytés L Gay-Escoda C. Pyostomatitis vegetans. Report of two cases and review of the literature. Oral surgery Oral medicine Oral pathology Oral radiology endodontics (2005) 99:447–54. doi: 10.1016/j.tripleo.2003.08.022 15772593

[36] Kumar KM Nachiammai N Madhushankari GS. Association of oral manifestations in ulcerative colitis: A pilot study. J Oral Maxillofac Pathol JOMFP (2018) 22:199–203. doi: 10.4103/jomfp.JOMFP_223_16 30158772 PMC6097373

[37] Muhvić-Urek M Tomac-Stojmenović M Mijandrušić-Sinčić B. Oral pathology in inflammatory bowel disease. World J Gastroenterol (2016) 22:5655–67. doi: 10.3748/wjg.v22.i25.5655 PMC4932203 27433081

[38] Femiano F Lanza A Buonaiuto C Perillo L Dell'Ermo A Cirillo N. Pyostomatitis vegetans: A review of the literature. Medicina oral patologia Oral y cirugia bucal (2009) 14:E114–7. 19242389

[39] Yasuda M Amano H Nagai Y Tamura A Ishikawa O Yamaguchi S. Pyodermatitis-pyostomatitis vegetans associated with ulcerative colitis: Successful treatment with total colectomy and topical tacrolimus. Dermatol (Basel Switzerland) (2008) 217:146–8. doi: 10.1159/000135708 18523389

[40] Werchniak AE Storm CA Plunkett RW Beutner EH Dinulos JGH. Treatment of pyostomatitis vegetans with topical tacrolimus. J Am Acad Dermatol (2005) 52:722–3. doi: 10.1016/j.jaad.2004.11.041 15793543

[41] Goldinova A Tan CX Bouma G Duijvestein M Brand HS de Boer NK. Oral health and salivary function in ulcerative colitis patients. United Eur Gastroenterol J (2020) 8:1067–75. doi: 10.1177/2050640620957138 PMC7724544 32878578

[42] Elahi M Telkabadi M Samadi V Vakili H. Association of oral manifestations with ulcerative colitis. Gastroenterol Hepatol bed to bench (2012) 5:155–60. PMC4017478 24834217

[43] Thrash B Patel M Shah KR Boland CR Menter A. Cutaneous manifestations of gastrointestinal disease: part II. J Am Acad Dermatol (2013) 68(2):211.e1–246. doi: 10.1016/j.jaad.2012.10.036 23317981

[44] Cui RZ Bruce AJ Rogers RS. Recurrent aphthous stomatitis. Clinics Dermatol (2016) 34:475–81. doi: 10.1016/j.clindermatol.2016.02.020 27343962

[45] Katsanos KH Torres J Roda G Brygo A Delaporte E Colombel JF. Review article: Non-malignant oral manifestations in inflammatory bowel diseases. Alimentary Pharmacol Ther (2015) 42:40–60. doi: 10.1111/apt.13217 25917394

[46] Jurge S Hegarty AM Hodgson T. Orofacial manifestations of gastrointestinal disorders. Br J Hosp Med (London Engl 2005) (2014) 75:497–501. doi: 10.12968/hmed.2014.75.9.497 25216165

[47] Rogler G Singh A Kavanaugh A Rubin DT. Extraintestinal manifestations of inflammatory bowel disease: Current concepts, treatment, and implications for disease management. Gastroenterology (2021) 161:1118–32. doi: 10.1053/j.gastro.2021.07.042 PMC8564770 34358489

[48] Khozeimeh F Shakerin H Daghaghzadeh H Najarzadegan F Golestannejad Z Adibi P. Oral manifestations in inflammatory bowel disease: A cross-sectional study in isfahan. Dental Res J (2021) 18:4. doi: 10.4103/1735-3327.310033 PMC8122690 34084291

[49] Habashneh RA Khader YS Alhumouz MK Jadallah K Ajlouni Y. The association between inflammatory bowel disease and periodontitis among jordanians: A case-control study. J periodontal Res (2012) 47:293–8. doi: 10.1111/j.1600-0765.2011.01431.x 22050539

[50] Laranjeira N Fonseca J Meira T Freitas J Valido S Leitão J. Oral mucosa lesions and oral symptoms in inflammatory bowel disease patients. Arq Gastroenterol (2015) 52:105–10. doi: 10.1590/S0004-28032015000200006 26039827

[51] Reamy BV Derby R Bunt CW. Common tongue conditions in primary care. Am Family physician (2010) 81:627–34. 20187599

[52] Li H Sun J Wang X Shi J. Oral microbial diversity analysis among atrophic glossitis patients and healthy individuals. J Oral Microbiol (2021) 13:1984063. doi: 10.1080/20002297.2021.1984063 34676060 PMC8526005

[53] Klein B Thoppay JR De Rossi SS Ciarrocca K. Burning mouth syndrome. Dermatologic Clinics (2020) 38:477–83. doi: 10.1016/j.det.2020.05.008 32892856

[54] Gurvits GE Tan A. Burning mouth syndrome. World J Gastroenterol (2013) 19:665–72. doi: 10.3748/wjg.v19.i5.665 PMC3574592 23429751

[55] Pilati S Bianco BC Vieira D Modolo F. Histopathologic features in actinic cheilitis by the comparison of grading dysplasia systems. Oral Dis (2017) 23:219–24. doi: 10.1111/odi.12597 27759902

[56] Klichowska-Palonka M Komsta A Pac-Kożuchowska E. The condition of the oral cavity at the time of diagnosis of inflammatory bowel disease in pediatric patients. Sci Rep (2021) 11:21898. doi: 10.1038/s41598-021-01370-8 34753969 PMC8578335

[57] Katz J Shenkman A Stavropoulos F Melzer E. Oral signs and symptoms in relation to disease activity and site of involvement in patients with inflammatory bowel disease. Oral Dis (2003) 9:34–40. doi: 10.1034/j.1601-0825.2003.00879.x 12617256

[58] Huang C Kugathasan S. Markers that differentiate early from late IBD. Digestive Dis (Basel Switzerland) (2012) 30:380–2. doi: 10.1159/000338130 22796800

[59] Hajishengallis G. Periodontitis: from microbial immune subversion to systemic inflammation. Nat Rev Immunol (2015) 15:30–44. doi: 10.1038/nri3785 25534621 PMC4276050

[60] She YY Kong XB Ge YP Liu ZY Chen JY Jiang JW. Periodontitis and inflammatory bowel disease: A meta-analysis. BMC Oral Health (2020) 20:67. doi: 10.1186/s12903-020-1053-5 32164696 PMC7069057

[61] Zhang Y Qiao D Chen R Zhu F Gong J Yan F. The association between periodontitis and inflammatory bowel disease: A systematic review and meta-analysis. BioMed Res Int (2021) 2021:6692420. doi: 10.1155/2021/6692420 33778080 PMC7981176

[62] Lorenzo-Pouso AI Castelo-Baz P Rodriguez-Zorrilla S Pérez-Sayáns M Vega P. Association between periodontal disease and inflammatory bowel disease: A systematic review and meta-analysis. Acta Odontologica Scandinavica (2021) 79:344–53. doi: 10.1080/00016357.2020.1859132 33370548

[63] Papageorgiou SN Hagner M Nogueira AV Franke A Jäger A Deschner J. Inflammatory bowel disease and oral health: Systematic review and a meta-analysis. J Clin periodontology (2017) 44:382–93. doi: 10.1111/jcpe.12698 28117909

[64] Schmidt J Weigert M Leuschner C Hartmann H Raddatz D Haak R. Active matrix metalloproteinase-8 and periodontal bacteria-interlink between periodontitis and inflammatory bowel disease? J Periodontology (2018) 89:699–707. doi: 10.1002/JPER.17-0486 29574823

[65] Vavricka SR Manser CN Hediger S Vögelin M Scharl M Biedermann L. Periodontitis and gingivitis in inflammatory bowel disease: A case-control study. Inflammation Bowel Dis (2013) 19:2768–77. doi: 10.1097/01.MIB.0000438356.84263.3b 24216685

[66] Koutsochristou V Zellos A Dimakou K Panayotou I Siahanidou S Roma-Giannikou E. Dental caries and periodontal disease in children and adolescents with inflammatory bowel disease: A case-control study. Inflammation Bowel Dis (2015) 21:1839–46. doi: 10.1097/MIB.0000000000000452 25985243

[67] Grössner-Schreiber B Fetter T Hedderich J Kocher T Schreiber S Jepsen S. Prevalence of dental caries and periodontal disease in patients with inflammatory bowel disease: A case-control study. J Clin periodontology (2006) 33:478–84. doi: 10.1111/j.1600-051X.2006.00942.x 16820035

[68] Tan CXW Brand HS Kalender B De Boer NKH Forouzanfar T de Visscher JGAM. Dental and periodontal disease in patients with inflammatory bowel disease. Clin Oral Investigations (2021) 25:5273–80. doi: 10.1007/s00784-021-03835-6 PMC8370899 33619633

[69] Brozek JL Akl EA Alonso-Coello P Lang D Jaeschke R Williams JW. Grading quality of evidence and strength of recommendations in clinical practice guidelines. Part 1 of 3. An overview of the GRADE approach and grading quality of evidence about interventions. Allergy (2009) 64:669–77. doi: 10.1111/j.1398-9995.2009.01973.x 19210357

[70] Said HS Suda W Nakagome S Chinen H Oshima K Kim S. Dysbiosis of salivary microbiota in inflammatory bowel disease and its association with oral immunological biomarkers. DNA Res an Int J Rapid Publ Rep Genes Genomes (2014) 21:15–25. doi: 10.1093/dnares/dst037 PMC3925391 24013298

[71] Xun Z Zhang Q Xu T Chen N Chen F. Dysbiosis and ecotypes of the salivary microbiome associated with inflammatory bowel diseases and the assistance in diagnosis of diseases using oral bacterial profiles. Front In Microbiol (2018) 9:1136. doi: 10.3389/fmicb.2018.01136 PMC5988890 29899737

[72] Molinero N Taladrid D Zorraquín-Peña I de Celis M Belda I Mira A. Ulcerative colitis seems to imply oral microbiome dysbiosis. Curr Issues In Mol Biol (2022) 44:1513–27. doi: 10.3390/cimb44040103 PMC9164047 35723361

[73] Surna A Kubilius R Sakalauskiene J Vitkauskiene A Jonaitis J Saferis V. Lysozyme and microbiota in relation to gingivitis and periodontitis. Med Sci monitor Int Med J Exp Clin Res (2009) 15:CR66–73. 19179970

[74] Chandan JS Thomas T. The impact of inflammatory bowel disease on oral health. Br Dental J (2017) 222:549–53. doi: 10.1038/sj.bdj.2017.318 28387295

[75] Atarashi K Suda W Luo C Kawaguchi T Motoo I Narushima S. Ectopic colonization of oral bacteria in the intestine drives T1 cell induction and inflammation. Sci (New York N.Y.) (2017) 358:359–65. doi: 10.1126/science.aan4526 PMC5682622 29051379

[76] Souto R Andrade AFB Uzeda M Colombo APVJ. Prevalence of" non-oral" pathogenic bacteria in subgingival biofilm of subjects with chronic periodontitis. Brazilian Journal of Microbiology (2006) 37:208–15. doi: 10.1590/S1517-83822006000300002

[77] Zawadzki PJ Perkowski K Starościak B Baltaza W Padzik M Pionkowski K. Identification of infectious microbiota from oral cavity environment of various population group patients as a preventive approach to human health risk factors. Ann Agric Environ Med AAEM (2016) 23:566–9. doi: 10.5604/12321966.1226847 28030924

[78] Rautava J Pinnell LJ Vong L Akseer N Assa A Sherman PM. Oral microbiome composition changes in mouse models of colitis. J Gastroenterol Hepatol (2015) 30:521–7. doi: 10.1111/jgh.12713 25180790

[79] Tomasello G Mazzola M Leone A Sinagra E Zummo G Farina F. Nutrition, oxidative stress and intestinal dysbiosis: Influence of diet on gut microbiota in inflammatory bowel diseases. Biomed papers Med Faculty Univ Palacky Olomouc Czechoslovakia (2016) 160:461–6. doi: 10.5507/bp.2016.052 27812084

[80] Tomasello G Mazzola M Jurjus A Cappello F Carini F Damiani P. The fingerprint of the human gastrointestinal tract microbiota: A hypothesis of molecular mapping. J Biol regulators homeostatic Agents (2017) 31:245–9. 28337900

[81] Hevia A Milani C López P Cuervo A Arboleya S Duranti S. Intestinal dysbiosis associated with systemic lupus erythematosus. mBio (2014) 5:e01548–14. doi: 10.1128/mBio.01548-14 PMC4196225 25271284

[82] Cappello F Rappa F Canepa F Carini F Mazzola M Tomasello G. Probiotics can cure oral aphthous-like ulcers in inflammatory bowel disease patients: A review of the literature and a working hypothesis. Int J Mol Sci (2019) 20(20):5026. doi: 10.3390/ijms20205026 PMC6834154 31614427

[83] Zdravkovic ND Jovanovic IP Radosavljevic GD Arsenijevic AN Zdravkovic ND Mitrovic SL. Potential dual immunomodulatory role of VEGF in ulcerative colitis and colorectal carcinoma. Int J Med Sci (2014) 11:936–47. doi: 10.7150/ijms.8277 PMC4113587 25076849

[84] Vasovic M Gajovic N Brajkovic D Jovanovic M Zdravkovaic N Kanjevac T. The relationship between the immune system and oral manifestations of inflammatory bowel disease: A review. Central-European J Immunol (2016) 41:302–10. doi: 10.5114/ceji.2016.63131 PMC5099388 27833449

[85] Aleksandra Nielsen A Nederby Nielsen J Schmedes A Brandslund I Hey H. Saliva interleukin-6 in patients with inflammatory bowel disease. Scandinavian J Gastroenterol (2005) 40:1444–8. doi: 10.1080/00365520510023774 16316893

[86] Lin Z Wang Z Hegarty JP Lin TR Wang Y Deiling S. Genetic association and epistatic interaction of the interleukin-10 signaling pathway in pediatric inflammatory bowel disease. World J Gastroenterol (2017) 23:4897–909. doi: 10.3748/wjg.v23.i27.4897 PMC5526760 28785144

[87] Xiao Y Wang X-Q Yu Y Guo Y Xu X Gong L. Comprehensive mutation screening for 10 genes in Chinese patients suffering very early onset inflammatory bowel disease. World J Gastroenterol (2016) 22:5578–88. doi: 10.3748/wjg.v22.i24.5578 PMC4917618 27350736

[88] Engelhardt KR Shah N Faizura-Yeop I Kocacik Uygun DF Frede N Muise AM. Clinical outcome in IL-10- and IL-10 receptor-deficient patients with or without hematopoietic stem cell transplantation. J Allergy Clin Immunol (2013) 131:825–30. doi: 10.1016/j.jaci.2012.09.025 23158016

[89] Bastos MF Lima JA Vieira PM Mestnik MJ Faveri M Duarte PM. TNF-alpha and IL-4 levels in generalized aggressive periodontitis subjects. Oral Dis (2009) 15:82–7. doi: 10.1111/j.1601-0825.2008.01491.x 18992018

[90] Figueredo CM Brito F Barros FC Menegat JSB Pedreira RR Fischer RG. Expression of cytokines in the gingival crevicular fluid and serum from patients with inflammatory bowel disease and untreated chronic periodontitis. J Periodontal Res (2011) 46:141–6. doi: 10.1111/j.1600-0765.2010.01303.x 20701671

[91] Enver A Ozmeric N Isler SC Toruner M Fidan C Demirci G. An evaluation of periodontal status and cytokine levels in saliva and gingival crevicular fluid of patients with inflammatory bowel diseases. J Periodontology (2022). doi: 10.1002/JPER.22-0065 PMC10083950 35665507

[92] Plemmenos G Evangeliou E Polizogopoulos N Chalazias A Deligianni M Piperi C. Central regulatory role of cytokines in periodontitis and targeting options. Curr medicinal Chem (2021) 28:3032–58. doi: 10.2174/0929867327666200824112732 32838709

[93] Brakenhoff LKPM van der Heijde DM Hommes DW Huizinga TWJ Fidder HH. The joint-gut axis in inflammatory bowel diseases. J Crohn's Colitis (2010) 4:257–68. doi: 10.1016/j.crohns.2009.11.005 21122514

[94] Vavricka SR Schoepfer A Scharl M Lakatos PL Navarini A Rogler G. Extraintestinal manifestations of inflammatory bowel disease. Inflammation Bowel Dis (2015) 21:1982–92. doi: 10.1097/MIB.0000000000000392 PMC4511685 26154136

[95] Sartor RB. Mechanisms of disease: Pathogenesis of crohn's disease and ulcerative colitis. Nat Clin Pract Gastroenterol Hepatol (2006) 3:390–407. doi: 10.1038/ncpgasthep0528 16819502

[96] Ficarra G Baroni G Massi D. Pyostomatitis vegetans: Cellular immune profile and expression of IL-6, IL-8 and TNF-alpha. Head Neck Pathol (2010) 4:1–9. doi: 10.1007/s12105-009-0149-7 20237982 PMC2825530

[97] Triantafillidis JK Vagianos C Papalois AE. The role of enteral nutrition in patients with inflammatory bowel disease: Current aspects. BioMed Res Int (2015) 2015:197167. doi: 10.1155/2015/197167 25793189 PMC4352452

[98] Hartman C Eliakim R Shamir R. Nutritional status and nutritional therapy in inflammatory bowel diseases. World J Gastroenterol (2009) 15:2570–8. doi: 10.3748/wjg.15.2570 PMC2691486 19496185

[99] Piskin S Sayan C Durukan N Senol M. Serum iron, ferritin, folic acid, and vitamin B12 levels in recurrent aphthous stomatitis. J Eur Acad Dermatol Venereology JEADV (2002) 16:66–7. doi: 10.1046/j.1468-3083.2002.00369.x 11952294

[100] Dignass AU Gasche C Bettenworth D Birgegård G Danese S Gisbert JP. European Consensus on the diagnosis and management of iron deficiency and anaemia in inflammatory bowel diseases. J Crohn's colitis (2015) 9:211–22. doi: 10.1093/ecco-jcc/jju009 25518052

[101] Sun A Lin H-P Wang Y-P Chiang C-P. Significant association of deficiency of hemoglobin, iron and vitamin B12, high homocysteine level, and gastric parietal cell antibody positivity with atrophic glossitis. J Oral Pathol Med Off Publ Int Assoc Oral Pathologists Am Acad Oral Pathol (2012) 41:500–4. doi: 10.1111/j.1600-0714.2011.01122.x 22188475

[102] Wu Y-C Wang Y-P Chang JY-F Cheng S-J Chen H-M Sun A. Oral manifestations and blood profile in patients with iron deficiency anemia. J Formosan Med Assoc = Taiwan yi zhi (2014) 113:83–7. doi: 10.1016/j.jfma.2013.11.010 24388269

[103] Lin H-P. Significant association of hematinic deficiencies and high blood homocysteine levels with burning mouth syndrome. J Formosan Med Assoc = Taiwan yi zhi (2013) 112:319–25. doi: 10.1016/j.jfma.2012.02.022 23787008

[104] Park KK Brodell RT Helms SE. Angular cheilitis, part 2: Nutritional, systemic, and drug-related causes and treatment. Cutis (2011) 88:27–32. 21877503

[105] Jajam M Bozzolo P Niklander S. Oral manifestations of gastrointestinal disorders. J Clin Exp dentistry (2017) 9:e1242–8. doi: 10.4317/jced.54008 PMC5694155 29167716

[106] Mizukami Y Imanishi H Tateishi C Kaneshiro S Sowa-Osako J Ohsawa M. Successful treatment of pyostomatitis vegetans with ulcerative colitis using dapsone without systemic steroids. J Dermatol (2019) 46:e316–7. doi: 10.1111/1346-8138.14885 30969436

[107] Bardasi G Romagnoli A Foschini MP Mantovani A Alvisi P. Pyostomatitis vegetans in a pediatric patient with ulcerative colitis: Case report of a rare pediatric inflammatory bowel disease extraintestinal manifestation and review of the literature. Eur J Gastroenterol Hepatol (2020) 32:889–92. doi: 10.1097/MEG.0000000000001723 32282544

[108] Kitayama A Misago N Okawa T Iwakiri R Narisawa Y. Pyodermatitis-pyostomatitis vegetans after subtotal colectomy for ulcerative colitis. J Dermatol (2010) 37:714–7. doi: 10.1111/j.1346-8138.2010.00961.x 20649713

[109] Jurge S Kuffer R Scully C Porter SR. Mucosal disease series. Number VI. recurrent aphthous stomatitis. Oral Dis (2006) 12:1–21. doi: 10.1111/j.1601-0825.2005.01143.x 16390463

[110] Pereira MS Munerato MC. Oral manifestations of inflammatory bowel diseases: Two case reports. Clin Med Res (2016) 14:46–52. doi: 10.3121/cmr.2015.1307 26864508 PMC4851452

[111] Aggarwal H Singh MP Nahar P Mathur H Gv S. Efficacy of low-level laser therapy in treatment of recurrent aphthous ulcers - a sham controlled, split mouth follow up study. J Clin Diagn Res JCDR (2014) 8:218–21. doi: 10.7860/JCDR/2014/7639.4064 PMC3972568 24701539

[112] Nijakowski K Rutkowski R Eder P Korybalska K Witowski J Surdacka A. Changes in salivary parameters of oral immunity after biologic therapy for inflammatory bowel disease. Life (Basel Switzerland) (2021) 11(12):1409. doi: 10.3390/life11121409 PMC8708388 34947940

[113] Cotti E Mezzena S Schirru E Ottonello O Mura M Ideo F. Healing of apical periodontitis in patients with inflammatory bowel diseases and under anti-tumor necrosis factor alpha therapy. J Endodontics (2018) 44:1777–82. doi: 10.1016/j.joen.2018.09.004 30390972

[114] Calobrisi SD Mutasim DF McDonald JS. Pyostomatitis vegetans associated with ulcerative colitis. temporary clearance with fluocinonide gel and complete remission after colectomy. Oral Surgery Oral Medicine Oral Pathology Oral Radiology Endodontics (1995) 79:452–4. doi: 10.1016/s1079-2104(05)80126-x 7614204

